# Combined Effect of Active Packaging of Polyethylene Filled with a Nano-Carrier of Salicylate and Modified Atmosphere to Improve the Shelf Life of Fresh Blueberries

**DOI:** 10.3390/nano10122513

**Published:** 2020-12-14

**Authors:** Valeria Bugatti, Maria Cefola, Nicola Montemurro, Michela Palumbo, Laura Quintieri, Bernardo Pace, Giuliana Gorrasi

**Affiliations:** 1 Department of Industrial Engineering, University of Salerno, Via Giovanni Paolo II, 132, 84084 Fisciano, Italy; vbugatti@unisa.it; 2 Nice Filler s.r.l., Via Loggia dei Pisani, 25, 80133 Napoli, Italy; 3 Institute of Sciences of Food Production, National Research Council of Italy (CNR), c/o CS-DAT, Via Michele Protano, 71121 Foggia, Italy; maria.cefola@ispa.cnr.it (M.C.); nicola.montemurro@ispa.cnr.it (N.M.); michela.palumbo@ispa.cnr.it (M.P.); 4 Institute of Sciences of Food Production, National Research Council of Italy (CNR), Via Amendola 122/O, 70125 Bari, Italy; laura.quintieri@ispa.cnr.it; 5 Department of Science of Agriculture, Food and Environment, University of Foggia, Via Napoli, 25, 71122 Foggia, Italy

**Keywords:** layered double hydroxides (LDHs), polyethylene, microbial control, postharvest quality, marketability

## Abstract

Blueberries are popular among consumers for their high nutritional value but are highly perishable due to the microbial decay. The use of active packaging that is able to interact with the food through releasing or absorbing substances can be a valid approach to preserve the quality and increase the fruit’s shelf-life. In this paper, an active packaging based on polyethylene (PE) filled with a nano-carrier of salicylate was prepared and characterized. Fresh blueberries were packaged in passive modified atmosphere packaging (pMA) for 13 days at 8 °C. The combination of the active filler in bulk and pMA showed a significant inhibition of mold development and a reduction of the respiration rate of fruits. Moreover, the release of salicylate on blueberries did not alter the fruits’ sensory traits and preserved the firmness and the nutritional quality. Finally, the combination of active packaging and pMA resulted a valid solution to extend blueberries’ shelf-life up to 13 days.

## 1. Introduction

Highbush blueberry (*Vaccinium corymbosum* L.), also called blue huckleberry [1,2], in the last years, has become popular in Europe, where the production and commercialization have extensively increased [3] for fresh consumption and its derived products [4]. The increase in popularity of these small fruits among consumers is mainly due to high nutritional value and their pleasing flavor and taste [5,6].

These fruits lose their shelf-life from gray mold (*Botrytis* spp.) development, mechanical damage, and water and nutritional loss [7,8,9].

Modified atmosphere (MA) packaging reducing the respiration rate and weight loss during storage might be a useful tool to prolong blueberry shelf-life. In addition to MA, active packaging represents an innovative approach to prolong the fruits’ shelf-life, ensuring their quality, integrity, and safety. The European regulation (EC) No 450/2009 defines an active packaging as a system that interacts with the food, releasing or absorbing substances into or from the packaged food or the environment surrounding the food. As a consequence, active packaging can be divided in two main categories: scavenging systems and active-releasing systems. The scavenging materials are able to remove undesired compounds from the food or its environment (i.e., moisture, oxygen, carbon dioxide, ethylene, odors), while the active-releasing materials add compounds to the packaged food or into the headspace (i.e., antimicrobial molecules, antioxidants, ethylene, carbon dioxide, flavors, ethanol) [10,11,12,13,14]. The addition of active molecules to the packaging, instead of directly to the food, helps to decrease the amount of such substances required to prolong the food’s shelf life. Indeed, when the active molecules are added directly to the product, their activity may be reduced or inhibited as a result of interaction between the active substances and the food components, and/or during the processing of the product. With the aim of introducing as few active molecules as possible into the bulk of the packaging, an interesting solution is the use of a nano-carrier that is able to release the molecules in a controlled way. In the last few years, it has been demonstrated that Layered Double Hydroxides (LDHs) are excellent carriers to delivery active molecules from the food packaging materials [15,16,17,18]. They can be produced on a large scale and with a high level of purity and can be modified with almost any type of organic anion. They improve the physical and barrier properties of the polymer matrix in which they are dispensed and act as nano-carriers of the active molecules intercalated between their layers.

The present paper reports the preparation and characterization of active packaging obtained using a master batch of polyethylene (PE) and 25% (wt/wt) of active filler based on LDH and salicylate (listed in EC-Directive 10/2011/EC of 14 January 2011) [19]. Then, the postharvest quality of fresh blueberries packaged under modified atmosphere using the produced active material was followed during the storage at 8 °C for 13 days.

## 2. Materials and Methods

### 2.1. Active Packaging Material Preparation

Polyethylene (PE) (PRISMA AD PE RIPE RET 91735), moisture 1500 ppm and density 0.92 g/cm^3^, used for the masterbatch and the film, was supplied by Frilvam SpA (Nerviano, Italy). The active filler, having the trade name of A3B9^®^ and based on an LDH intercalated with antimicrobial salicylate anion (Commission Regulation (EU) No. 10/2011), was produced by Nicefiller Ltd. (Fisciano, Italy), which is a startup of the University of Salerno (Salerno, Italy). The synthesis was conducted accordingly to a previously reported procedure [20]. The filler was micronized by Food and Phama Systems srl (Fiorenzuola d’Arda, Italy) to obtain 1–2 micron sized particles. The PE masterbatch (MB) was obtained by mixing 25 wt% of A3B9^®^ filler, previously dried at 105 °C for 24 h, with 75 wt% of PE in a twin-screw extruder at 110 °C and extruding the mixture through a strand die. The strand was cooled, cut, and dried at 70 °C for 3 h.

Starting from 40% of MB based on PE and 25% filler A3B9^®^, rolls (10–12 cm wide) of multilayer PE film (three layers) were prepared using a three-extruder bubble co-extrusion plant, with 10% filler in the 10 micron thick PE layer (50-micron total thickness). The operation determined the process conditions in terms of thermal profiles along the machines, screw rotation speed, collection and inflation and ironing conditions. A sample of PE with the active molecule, at the same amount present in the active filler (i.e., 8.4%), simply mixed into the film was prepared in the same described experimental conditions. Then, active bags were prepared from active PE coils and used to package blueberries as described below.

### 2.2. Active Packaging Material Characterization

The masterbatch was prepared by means of an ICMA San Giorgio (San Giorgio su Legnano, Italy) co-rotating twin-screw extrusion system, with a temperature profile varying between 110 and 120 °C. The PE film was prepared by means of a Gefran Beyond Technology (Provaglio d’Iseo, Italy) three-extruder bubble co-extrusion plant, consisting of 3 extrusion lines for polyethylene film for the production of open mouth bags. There are 3 extruders, 60 Kw each, which are equipped with the towing and collection, respectively: two 800 mm and one 1000 mm.

X-ray diffraction (XRD) patterns were taken in reflection with an automatic Bruker diffractometer D8 (Karlsruhe, Germany), using nickel-filtered Cu Kα radiation (Kα = 1.54050 Å) and operating at 40 kV and 40 mA, with a step scan of 0.05° of 2θ and 3 s of counting time.

The release kinetics of salicylate was followed using a Shimadzu UV-2401 PC spectrometer (Kyoto, Japan). The tests were performed using 4 cm^2^ rectangular specimens placed into 25 mL Ethanol 50% solution and stirred at 100 rpm in an orbital shaker (VDRL MOD. 711+, Asal S.r.l., Milan, Italy). The release medium was withdrawn at fixed time intervals and replenished with fresh medium. The considered band was 230 nm.

Global migration tests were performed on the PE film treated with A3B9^®^ filler samples according to the following procedure. PE film specimens with 1 dm^2^ of surface area (10 cm × 10 cm, 0.10 mm thickness) were put into contact with 100 mL simulant (preconditioned at 40 °C) in a borosilicate glass tube closed with a screw cap internally layered with Teflon^®^. The obtained surface/volume ratio was 10 dm^2^/L. Migration tests after contact for 10 days at 40 °C were performed using as simulants A (Ethanol at 10%), B (Acetic acid at 3%), C (Ethanol at 20%), D1 (Ethanol at 50%), and D2 (oil). The overall migration test was performed on different aliquots from the same contact sample. The overall migration results were calculated by using 6 dm^2^/kg food (6 dm^2^/L simulant) as per the conventional EU surface/volume ratio. A known aliquot of the simulant from the contact solution was transferred into a weighted quartz capsule and evaporated to dryness until constant weight. From the differences between the weights, the overall migration was derived in accordance to EN 1186 Migration Testing for Food Contact Materials. The data were averaged on five samples.

### 2.3. Blueberries Packaging and Storage

Blueberries (*Vaccinium corymbosum* L., cv Rebel) were harvested at commercial maturity stage (full color berries and total soluble solids of 9.4 ± 1.1°Brix) by Naturagri srl (San Giorgio Lucano, Italy) and transported in refrigerated condition to the Postharvest Laboratory of Institute of Science of Food Production. About 4 kg of berries were selected to eliminate fruits with evident defects or diseases; then, 500 g of fruit were used for the initial analysis in three replicates, while the remaining samples were divided in 2 lots (one for each treatment) of about 1.5 kg each. Fruits (about 80 g for replicates) were put in PE active bags (15 × 10 cm) and closed in triplicate in a passive modified atmosphere (Active-pMA) or were put in open PE bags (Control). All samples were stored at 8 °C and were analyzed after 3, 7, and 13 days. Daily, the gas composition inside Active-pMA bags was measured using a gas analyzer (CheckPoint O_2_/CO_2_-Dansensor^®^ Mocon, Ringsted, Denmark). At each storage time, berries belonging to each packaging treatment, in triplicate, were analyzed for postharvest quality evaluation.

### 2.4. Blueberries Postharvest Quality Evaluation

#### 2.4.1. Chemicals and Reagents

For the quality determinations, methanol, acetone, 2,2-diphenyl-1-picrylhydrazyl (DPPH), 6-hydroxy-2,5,7,8-tetramethylchroman-2-carboxylic acid (Trolox), and gallic acid were obtained from Sigma–Aldrich (St. Louis, MO, USA). Folin–Ciocalteu’s phenol reagent was purchased from Merck (Darmstadt, Germany). For the microbiological analyses, all culture media and related supplements were purchased from Oxoid (OxoidSPA Rodano, Milano, Italy).

#### 2.4.2. Respiration Rate and Weight Loss

The respiration rate was measured initially and at each sampling time (just after the opening of the bags), using a closed system [21], placing 50 g of product (3 replicates per treatment) into 3.6 L sealed plastic jars (one jar for each replicate), where CO_2_ was allowed to accumulate. The CO_2_ concentration inside the jars was monitored at regular time intervals up to reach the value of 0.1% as the concentration of the CO_2_ standard. Respiration rate was evaluated at 8 °C and expressed as mL CO_2_/kg h. The weight loss was measured as reported in [22].

#### 2.4.3. Sensory Evaluation and Color

Berries were sensory scored by a group of eight trained researchers at the beginning of the experiment and during the storage period. A selected group of 10 panelists (made up of 5 females and 5 males), previously involved as members of trained descriptive analysis panels, was trained to illustrate the descriptors of blueberries.

Coded (3 digits) samples were presented to the trained researchers (judges) individually, to enable them to make independent evaluations. Off-odor (just after opening the bag) was evaluated using a 5-point scale where 5 = severe (very strong off-odor), 3 = moderate off-odor, and 1 = none (no off-odor) [23]. Visual quality was evaluated using a 5-point rating scale, where 5 = excellent and 1 = severe visual defects, according to [24].

The color was acquired using a colorimeter (CR-400-Konica Minolta, Osaka, Japan) as reported in Pace et al. [25] on 10 berries, as the mean value of two opposite point for each berry. The color was expressed as hue angle (h = arctan $\frac{b^{*}}{a^{*}}$) calculated from primary L*, a*, and b* readings.

#### 2.4.4. Firmness, Total Soluble Solids, Titratable Acidity, and pH

The berries firmness was evaluated on 10 fruits per replicate by using a texture analyzer (ZwickLine Z0.5-Zwick/Roell, Ulm, Germany) equipped with a plate of 100 mm in diameter. Results were expressed as the ratio between the force that achieved the 2 mm of deformation of the berries and the fruit weight (N/g).

For the measurement of total soluble solids, titratable acidity, and pH, the same berries extract was used. About 4 g of berries were homogenized (T 25 digital ULTRA-TURRAX^®^-IKA, Staufen, Germany) in 20 mL of distilled water for 1 min and then filtered through 2 layers of cheesecloth to obtain the berries extract. The total soluble solids content was measured using a digital refractometer (DBR35-XS Instruments, Carpi, Italy) and expressed in °Brix. Titratable acidity was determined using a semiautomatic titrator (PH-Burette 24 -Crison Instrument, Barcelona, Spain) with 0.1 M NaOH to the final pH 8.1, and results were expressed as percentage of citric acid. The same instrument was used to measure the pH.

#### 2.4.5. Total Phenols, Antioxidant Activity, and Carotenoids

The total phenols and antioxidant activity was measured in a previous study [26]. The content of total phenols was calculated based on the calibration curve of gallic acid (50–500 µg/mL, R^2^ = 0.99) and was expressed as mg gallic acid per 100 g of fresh weight (fw). For antioxidant activity, results were expressed as milligrams of Trolox per 100 g of fw using a Trolox calibration curve (82–625 µM).

For the determination of carotenoids, 5 g of berries tissue were homogenized in acetone/water (80:20 v/v) and then centrifuged at 6440× *g* for 5 min. To remove all pigments, the extraction procedure was repeated 3 times, and the extracts were combined. The absorbance (Abs) was read immediately after the extraction at three wavelengths: 663.2, 646.8, and 470 nm. Carotenoids were calculated using the following formula: [(1000 × Abs 470) − (1.82 × Ca) − (85.02 × Cb)]/198, where Ca = (12.25 × Abs 663.2) − (2.79 × Abs 646.8), and Cb = (21.50 × Abs 646.8) − (5.10 × Abs 663.2) [27]. Results were expressed as mg of carotenoids per 100 g of fruit on fw.

#### 2.4.6. Microbiological Analysis

At 0, 3, 7, and 13 days of cold incubation (8 °C), blueberry samples, in Active-pMA or Control, were transferred (25 g), in triplicate, into individual sterile stomacher bags and then homogenized in 225 mL of 0.1% peptone water for 1 min. Then, serial dilutions, in triplicate, of the resultant suspensions were performed in 0.1% peptone water and plated on selective culture media as follows: total aerobic bacterial counts (TBC) were enumerated on Plate Count Agar (PCA) supplemented with cycloheximide (0.1 mg/mL) after 24 h of incubation at 30 °C [28]; total coliforms were enumerated on Violet Red Bile Agar (VRBA) after 24 h at 37 °C [29]; *Pseudomonas* spp. were enumerated on Pseudomonads Agar Base (PSA; amended with *Pseudomonas* CFC selective supplement, Oxoid) after incubation at 30 °C for 24 h; yeasts and molds were enumerated on Dichloran Rose Bengal Chloranphenicol agar (DRBC, Merck, Germany) agar after 96 h at 25 °C [30]. Microbial counts were expressed as log CFU (colony-forming units) per gram of fresh blueberries.

#### 2.4.7. Statistical Analysis

For the postharvest quality parameters, the effect of treatments (Active-pMA and Control), storage time (3, 7, and 13 days), and their interaction were tested by performing a multifactor ANOVA using StatGraphics Centurion XVI.I (StatPoint Technologies, Inc., Warrenton, VA, USA). When the interaction (treatment *x* storage time) was significant, data were presented as graphs with mean values ± standard deviation. For microbiological analysis, statistical significance (*p* values) of the results was calculated by unpaired two-tailed Student’s *t* test using GraphPad Prism 8 software (San Diego, CA, USA).

## 3. Results

### 3.1. Packaging Material Characterization

**Figure 1:**
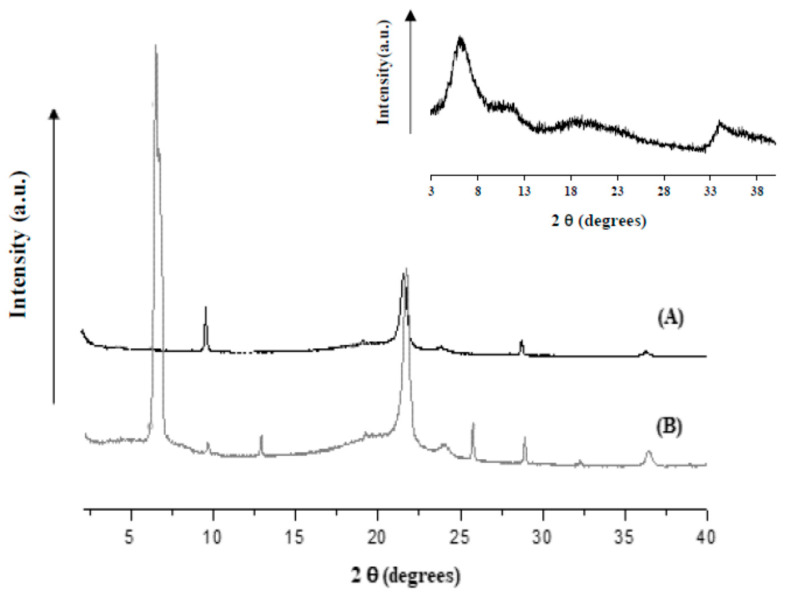
X-ray diffraction of unfilled polyethylene (PE) (**A**) and active film (**B**). Inset: Layered Double Hydroxides modified with salicylate molecule.

**Figure 2:**
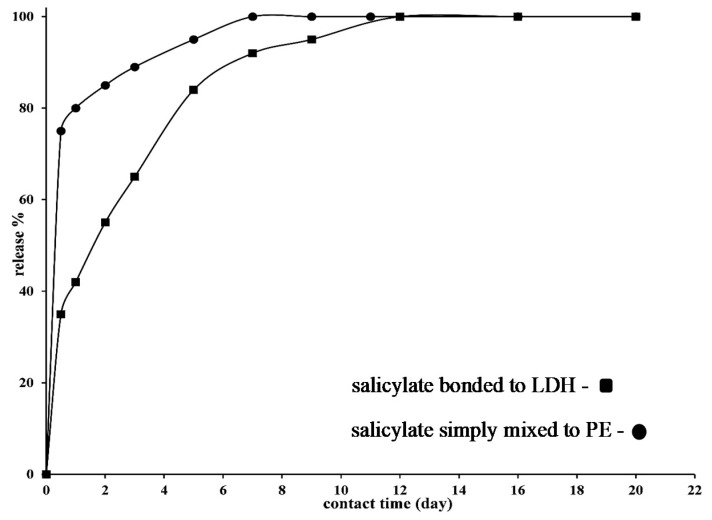
Release of salicylate bonded to Layered Double Hydroxides nanofiller from active film and simply blended into PE

Figure 1 reports the XRD of PE (A) and PE filled with LDH salicylate (B). It is evident that PE (Figure 1A) shows its typical orthorombic cell with the peaks at 2θ = 21.6° and 2θ = 23.8°. Such a structure is retained in the active film (Figure 1B). In the latter, there is also a peak at 2θ = 6.02° that is related to the intercalation of the active molecule (see inset) between the pristine LDH–NO_3_ layers for which the interlayer distance shows a peak at 2θ = 10.2° [31]. Figure 2 reports the release of salicylate from the active packaging as a function of the time and the release of the active molecule, at the same amount of the molecule intercalated between the LDHs’ layers (i.e., 8.4% wt/wt) simply blended into the PE film. After a first burst, due to the release of the active molecule from the film surface, it is possible to observe slower release zones. In the case of the sample with an active molecule simply blended to the PE, the second release step is due to the counter-diffusion of the salicylate from the bulk of the material. The complete release (100% of blended molecule) is reached in 7 days. The sample of active PE (PE filled with LDH and salicylate) shows three release zones. After the burst that is much lower than the one observed from the PE with the molecule simply blended, we observed two release steps in which the active molecule is de-intercalated from the LDHs’ lamellae and counter-diffused out of the bulk. The amount of released salicylate, at any contact time, is always slow for the active film. The complete release of the intercalated molecule is reached in 12 days. We underline that such measurements were used mainly to demonstrate that salicylate was successfully intercalated between the LDH’s layers. The migration of such molecules, being non-volatile, into the packaged fruits is supposed to not follow the same mechanism registered in saline solution. Anyway, blueberries are fruits with a “complex composition”, and at the interface fruit/packaging we do not exclude an ionic exchange of the active salicylate with anions inside the fruits.

In order to demonstrate that the prepared active packaging is suitable for food contact, we performed overall migration tests on the film treated. Table 1 reports the global migration evaluated on the active packaging, in different food simulants, accordingly to UNI EN 1186-1:2003 [32] and UNI EN 1186-9:2003 [33]. The experimental results, in compliance with the migration limits, demonstrate the suitability of the considered material for food contact.

### 3.2. Effect of Active-pMA on Postharvest Quality Parameters of Blueberries

**Figure 3:**
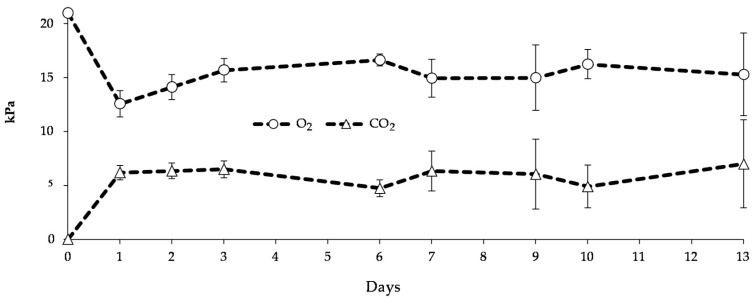
Changes in gas composition inside Active-pMA bags of blueberries (cv Rebel) stored for 13 days at 8 °C. Data are mean values (*n* = 3) ± standard deviation. pMA: passive modified atmosphere packaging.

Changes in gas composition inside Active-pMA bags are reported in Figure 3. Starting from the air composition (21 kPa O_2_ and 0.03 kPa CO_2_), the oxygen was gradually consumed by the product, with a consequent CO_2_ accumulation, reaching the equilibrium already after 24 h of storage at 8 °C, with mean values of 15.1 (±1.3) kPa O_2_ and 6.0 (±0.8) kPa CO_2_ (Figure 3).

The effect of treatments, storage time, and their interaction on the quality parameters evaluated in blueberries is reported in Table 2. Respiration rate, weight loss, and visual quality were affected by both treatments and storage time, and by their interaction. In addition, firmness was affected only by the interaction treatment x storage time. Regarding the other quality parameters, such as color, off-odor, total soluble solids, titratable acidity, pH, total phenols, antioxidant activity, and total carotenoids, the multifactor ANOVA analysis did not showed any significant effect of the factors considered. Regarding the parameters just mentioned, their values at harvest are reported in Table 3. No off-odor was perceived by judges during the trial.

**Figure 4:**
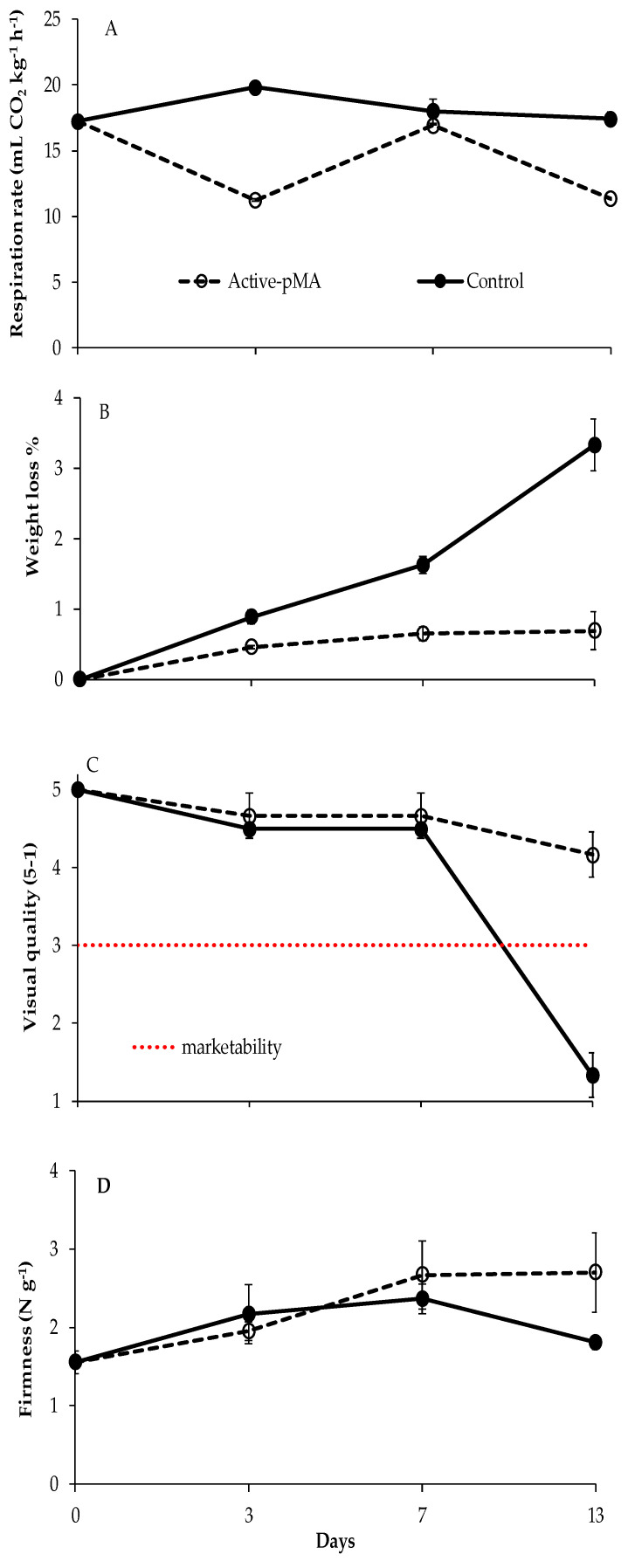
Changes in respiration rate (**A**), weight loss (**B**), visual quality (**C**) and firmness (**D**) in blueberries (cv Rebel) stored in Active-pMA or in air (Control) for 13 days at 8 °C. Data are mean values (*n* = 3) ± standard deviation.

Changes in the respiration rate, weight loss, visual quality, and firmness during storage are reported in Figure 4. The respiration rate measured at harvest was 17.2 (± 0.3) mL CO_2_/kg h, and this value remained unchanged in the Control for the entire experiment. Meanwhile, in Active-pMA, a significant reduction was observed after 3 days followed by an increase after 7 days, and a slight decrease at the end of the storage was measured (Figure 4A). As expected, samples stored in the Control showed a significant weight loss during storage, reaching a mean value of 3.3 (±0.4)% after 13 days at 8 °C, while in Active-pMA, the weight loss was very restricted (0.7 ± 0.3%) (Figure 4B).

Regarding the visual quality, as shown in Figure 4C, the blueberries stored in Active-pMA were judged very good (mean score of about 4.5) for the entire storage, whereas Control samples showed a significant reduction of visual quality starting from the 7th day in storage, reaching at the end of the trial a mean score below 2 (Figure 4C). This was due to the dehydration and to the mold development observed on the Control fruit. As for firmness, at the end of storage, the Control fruits were softer than the Active-pMA fruits (Figure 4D).

**Figure 5:**
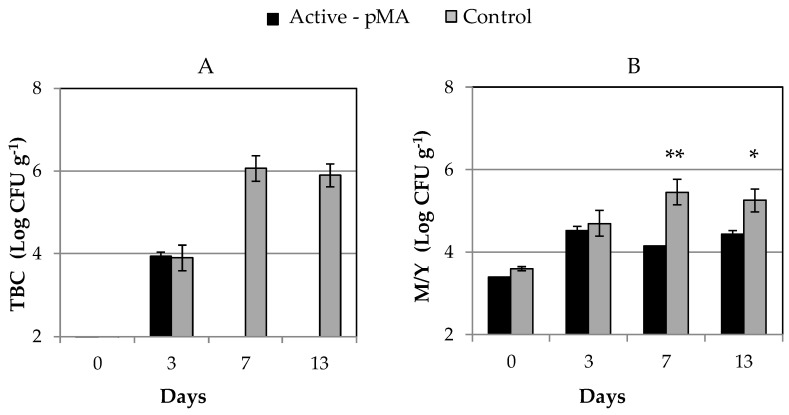
Total bacteria counts (TBC) (**A**), mold and yeast (M/Y) (**B**) (Log colony-forming units (CFU) g^−1^) determined in blueberries (cv.Rebel) stored in Active-pMA or in air (Control) for 13 days at 8 °C. Data are mean values (*n* = 3) ± standard deviation. Asterisks indicate statistically significance differences (Student’s test, ** *p* < 0.01 * *p* < 0.05).

Results from microbiological analysis showed the inhibitory effect of Active-pMA against all microbial populations naturally contaminating blueberries and herein analyzed (TBC, *Pseudomonas* spp, total coliforms, yeast, and molds). Figure 5 shows TBC, yeast, and mold counts registered in samples stored in Active-pMA and the related Control throughout cold storage.

In particular, mold and yeast reduced significantly (*p* < 0.05) their load in the Active-pMA sample starting from the 7th day of incubation. Likewise, except for day 3 of sampling, TBC in Active-pMA blueberries did not show concentration values above the detection limit (2 log CFU/g) over the entire period of incubation; by contrast, in the Control sample, TBC reached about 6 log CFU/g at the end of storage. Regarding *Pseudomonas* spp. and total coliforms, counts of 4 and 6 log CFU/g, respectively were registered in Control samples at the end of the storage; no bacterial cells were instead enumerated in the Active-pMA fruits (above the detection limit).

For the visual quality evaluation, the following 5-point rating scale was used: 5 = excellent (fresh appearance, full sensory acceptability); 4 = good (product acceptable from a sensory point of view); 3 = limit of sensory acceptability; 2 = product has notable visual defects; and 1 = severe visual defects.

## 4. Discussion

In the present research paper, the combined effect of active bags and passive modified atmosphere packaging (pMA) of 15 kPa O_2_ and 6 kPa CO_2_ at equilibrium on the quality of blueberries stored until 13 days at 8 °C was investigated. Since then, several researchers reported the fungistatic action of CO_2_ at concentrations higher than 10 kPa [34,35,36,37]; the effect on the reduction of the molds development in blueberries stored in Active-pMA was probably due to the use of antimicrobial packaging rather than the CO_2_ level inside the bags. In support of this hypothesis, the results showed that the antimicrobial effect was registered starting from the 7th day of storage, a sampling time in which the salicylate concentration was released by more than 80%. The efficacy of salicylic acid used as dipping solution in combination or not with heat and ultrasound treatments, or incorporated in edible coating, in counteracting microbial spoilage was previously reported for several stored fruits [38,39,40]. In accordance with our results, the authors reported that salicylic acid effectively had delayed the development of molds by maintaining fruit firmness and inner quality [40,41]. Recently, [42] found a significant effect of an active packaging based on salycilate release on the reduction of total mesophilic aerobic count (TBC) and mold and yeast population in table grapes stored for 14 days at 10 °C.

In detail, the mechanism of action involved conidia membrane damage with the consequent inhibition of fungal spore germination [43]. In addition, to molds contamination, the occurrence of several bacteria (e.g., *coliforms*, *Listeria* spp.) also contribute to blackberries decays within several days after harvest [44,45,46]. In particular, coliform contamination could be influenced by the conditions of hygiene prevailing during post-harvest handling and packing [47]. Interestingly, the antimicrobial effect of the herein developed packaging system was registered also against this latter population, thus improving hygiene standards of fruits during storage.

On the other hand, the atmosphere modification causes a significant reduction of respiration rate as compared with the Control. This behavior was previously observed in other produce [22,48], and, in general, it is well known that the modification of the atmosphere is able to reduce the rate of respiration of fruit and vegetables [49].

Blueberries postharvest quality loss is mainly due to mold development and dehydration, which results in fruit shriveling, brightness loss, and softening. All these factors affect the visual quality, limiting the marketability [50]. The use of Active-pMA was able to keep a good relative humidity inside the bags, avoiding water loss, maintaining the firmness, and thus preserving the fruit visual quality. The authors of [51] reported that in order to delay softening during the postharvest life of blueberries, it is important minimize the weight loss and the cell wall degradation. These results were in accordance with previous studies exploiting salicylic acid as a preservative agent of stored fruits [38,39]. Moreover, no off-odor was perceived in blueberries stored in active bags; thus, the salicylate release did not alter the fruit sensory quality.

Regarding the compositional quality, the Active-pMA preserved the initial content in phenolic compounds, total carotenoids, and the antioxidant activity during storage. Blueberries are fruit with one of the highest antioxidant capacities that is largely influenced by total phenolic content and in a small portion by ascorbic acid [52,53]. Starting from these points, maintaining a high level of phenolic compounds and antioxidant activity is a priority for the postharvest handling process.

## 5. Conclusions

A novel food packaging material based on polyethylene coated with a food grade acrylic resin filled with a nano-carrier of antimicrobial salicylate (listed in EC-Directive 10/2011) was prepared. The nano-carrier was constituted of an anionic clay, such as layered double hydroxide, to which the active molecule has been linked with an ionic bond. The successful incorporation of the antimicrobial into the layered double hydroxide was demonstrated through X-ray diffraction analysis and the study of controlled release.

The use of Active-pMA resulted in valid solution to store blueberries for 13 days at 8 °C, allowing a 50% shelf-life extension respect to the Control. The main effect of Active-pMA was the control of mold development, which was mainly due to the release of antimicrobial agents. Moreover, the storage in active bags delayed the rate of respiration and limited weight loss, preserving the fruit firmness. The Active-pMA also preserved the content in healthy compounds. In support of this hypothesis, the results showed that the antimicrobial effect was registered starting from the 7th day of storage, a sampling time in which the salicylate concentration was released by more than 80%.

## Figures and Tables

**Table 1:** Global migration into aqueous food simulant by filling a container UNI EN 1186-1: 2003 + UNI EN 1186-9: 2003 for the film based on the active PE filled with Layered Double Hydroxides and salicylate (contact time 10 day, temperature test 40 °C).

Simulant		Global Migration Average in the Simulant (mg/dm^2^)
A	Ethanol at 10% (*v/v*)	2.1
B	Acetic acid at 3% (*v/v*)	4.2
C	Ethanol at 20% (*v/v*)	2.5
D1	Ethanol at 50% (*v/v*)	3.7
D2	Oil	<2
Limit		10

**Table 2:** Effect of treatment (Active-pMA and Control) (A), storage time (3, 7, and 13 days at 8 °C) (B) and their interaction (A × B) on quality parameters evaluated in blueberries (cv Rebel).

Quality Parameter	A	B	A × B
Respiration rate (mL CO_2_/kg h)	****	****	****
Weight loss %	****	****	****
Off-odor (5-1)	ns	ns	ns
Visual quality (5-1)	****	****	****
Color (hue angle)	ns	ns	ns
Firmness (N/g)	ns	ns	*
Total soluble solids (°Brix)	ns	ns	ns
Titratable acidity (citric acid %)	ns	ns	ns
pH	ns	ns	ns
Total phenols (mg gallic acid/100 g fw)	ns	ns	ns
Antioxidant activity (mg Trolox/100 g fw)	ns	ns	ns
Carotenoids (mg/100 g fw)	ns	ns	ns

Asterisks indicate the significance level for each factor of the ANOVA test (ns, not significant; * *p* ≤ 0.05; **** *p* ≤ 0.0001).

**Table 3:** Quality parameters evaluated in blueberries (*Vaccinium corymbosum* L., cv *Rebel*) at harvest. Data are mean of three replicates ± standard deviation.

Quality Parameter			
Total soluble solids (°Brix)	10.0	±	0.4
pH	4.5	±	0.3
Hue angle (h°)	106.6	±	6.3
Total phenols (mg gallic acid/100 g fw)	222.4	±	29.3
Antioxidant activity (mg Trolox/100 g fw)	216.1	±	27.5
Titratable acidity (citric acid %)	1.19	±	0.10
Carotenoids (mg/100 g fw)	0.52	±	0.19
